# Treatment of Neglected Elbow Dislocation with Secondary Heterotopic Ossification

**DOI:** 10.3390/diseases13110369

**Published:** 2025-11-11

**Authors:** Mihai Tudor Gavrilă, Vlad Cristea, Cristea Stefan

**Affiliations:** 1 Emergency Hospital Saint Pantelimon Bucharest, 021659 Bucharest, Romania; 2 Hospital Colentina Bucharest, 020125 Bucharest, Romania; vladcristea@hotmail.com

**Keywords:** elbow dislocation, stiffness, heterotopic ossification, radial fracture, arthroplasty, posterior approach

## Abstract

A traumatic elbow dislocation that remains unreduced for more than three weeks is considered a neglected elbow dislocation. We report a case of a patient with a neglected elbow dislocation combined with a terrible triad injury (elbow dislocation with fractures of the coronoid process and radial head). Initially, the patient was managed with three weeks of cast immobilization followed by physiotherapy. However, six months after the trauma, he presented to our clinic with severe heterotopic ossification, significant pain, and nearly complete elbow stiffness. An open surgical intervention was performed, involving excision of the heterotopic bone, reduction in the dislocation, and suturing of the anterior capsule to the coronoid process. Given the irreparable fracture of the radial head, radial head arthroplasty was also performed. At 18-month follow-up, the elbow was stable and pain-free, with flexion–extension of 80°, pronation of 85°, and supination of 80°. This case underscores the critical importance of early diagnosis and intervention to prevent long-term complications in neglected elbow dislocations.

## 1. Introduction

Elbow dislocation is the second most common major joint dislocation in adults, following that of the shoulder [1]. These injuries frequently involve not only ligamentous disruption but also associated fractures, which, if not promptly recognized and adequately treated, may lead to significant long-term functional impairment. In many instances, the presence of intra-articular loose fragments renders simple closed reduction impossible. Dislocations that remain unreduced for more than three to six weeks are classified as neglected elbow dislocations [2].

According to the literature, neglected elbow dislocations are typically associated with severe functional deficits, including joint stiffness, and often require open surgical intervention to restore motion and stability [2,3,4,5]. Management of these chronic cases is particularly challenging due to extensive soft-tissue contractures, ligamentous insufficiency, fibrosis, potential nerve involvement, myositis ossificans, patient noncompliance, and the necessity for prolonged postoperative rehabilitation. The principal surgical objective is to achieve a stable, pain-free, and functional elbow with proper joint congruency.

In the present case, the patient sustained a complex injury known as the “terrible triad”, characterized by a posterior elbow dislocation combined with fractures of the radial head and the coronoid process. The patient presented with pain, deformity in extension, and a markedly restricted range of motion that severely limited daily activities. Repeated attempts at mobilization contributed to progressive stiffness and the development of heterotopic ossification. The management of such injuries carries a high risk of complications, including neurovascular injury, and the functional outcome can be unpredictable.

Elbow dislocations are classified according to the direction of ulnar displacement relative to the humerus. The main types include posterior (further subdivided into posterolateral and posteromedial), anterior, medial, and lateral dislocations. Among these, posterior dislocations are the most frequent, accounting for approximately 90% of cases [6]. They typically occur following a fall onto an outstretched hand, resulting in a posteriorly directed force transmitted through the forearm. Dislocations are termed simple when no fracture is present and complex when associated with fractures of the radial head, coronoid process, or ligamentous disruption, as observed in the present case.

During such injuries, valgus stress initially compromises the medial collateral ligament, which is often the first structure to fail [7]. As the mechanism progresses, the lateral collateral ligament complex may also be disrupted, further destabilizing the joint. The combination of valgus stress and hyperextension produces posterior and lateral displacement of the proximal radius and ulna. Hyperextension also disengages the coronoid process from the trochlea, often resulting in avulsion fragments. This constellation of injuries defines the terrible triad, consisting of elbow dislocation, radial head fracture, and coronoid fracture. Furthermore, the anterior capsule and adjacent musculature may tear, exacerbating instability. Consequently, the elbow typically dislocates posteriorly or posterolaterally, depending on the magnitude and direction of the applied forces [7].

During the traumatic event, the radial head frequently impacts the capitellum, causing comminution or displacement of intra-articular fragments [8]. These loose fragments often impede closed reduction, necessitating surgical intervention for anatomical restoration. In the present case, the initial injury was not managed surgically, and no attempt was made to perform open reduction or fragment removal.

Over time, the surrounding soft tissues—including the capsule, ligaments, and periarticular muscles—underwent progressive contracture, contributing substantially to stiffness and deformity [2,3]. Prolonged immobilization further exacerbated fibrosis within periarticular structures. When rehabilitation was eventually attempted, the elbow remained dislocated and severely restricted due to soft-tissue shortening and adhesion formation. Residual bone and cartilage fragments, combined with persistent inflammation, acted as local stimuli for heterotopic ossification and myositis ossificans. The resulting ectopic bone formation further limited motion and increased pain, rendering subsequent management increasingly complex.

Heterotopic ossification represents a significant complication following traumatic elbow injuries, particularly those involving fracture dislocations. The literature provides limited evidence regarding the specific risk factors contributing to the development of heterotopic ossification in such cases. The coexistence of a Terrible Triad injury with secondary heterotopic ossification, as observed in our patient, adds a distinctive and exceptional feature to this case, further highlighting its clinical relevance and complexity.

## 2. Patient Information

The patient was a 67-year-old right-handed man with an otherwise unremarkable medical and surgical history, aside from mild, well-controlled hypertension. He had no prior surgical procedures. At presentation, he reported persistent pain, elbow deformity, and severe stiffness that had gradually developed over six months following the initial injury. These symptoms significantly impaired his daily activities, particularly affecting flexion–extension and pronation–supination of the forearm. No relevant family or genetic history was noted.

## 3. Clinical Findings

**Figure 1:**
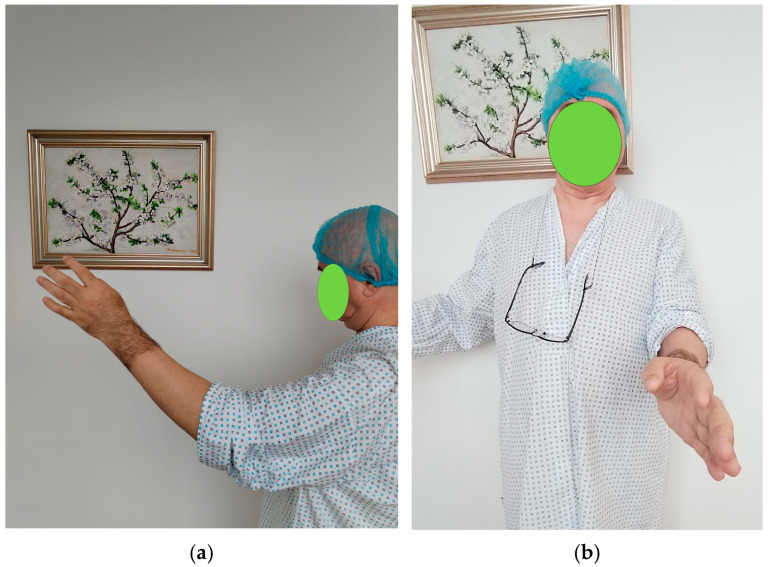
Clinical preoperative presentation (**a**) flexion–extension stiffness, (**b**) pronation–supination stiffness. The elbow was fixed in this position due to stiffness. Photographs were obtained with the patient’s written informed consent for scientific publication.

On clinical examination, the patient’s elbow was visibly deformed and rigid. The anterior aspect of the distal humerus was prominently palpable, while the olecranon was abnormally projecting posteriorly. There was notable retraction and shortening of the biceps muscle, likely due to prolonged disuse and altered joint mechanics. The elbow was fixed in a semi-flexed position of approximately 45–50°, allowing only minimal passive movement (about 5°). Active range of motion was nearly absent, and any attempt at joint movement caused pain. Both forearm rotation movements—pronation and supination—were completely restricted. Despite these functional limitations, there were no signs of distal neurovascular compromise: peripheral pulses were intact, capillary refill was normal, and motor and sensory testing of the radial, median, and ulnar nerves was unremarkable (Figure 1).

## 4. Timeline

The timeline below outlines the sequence of clinical events, diagnostic assessments, surgical interventions, and follow-up milestones for this case (Table 1).

## 5. Diagnostic Assessment

**Figure 2:**
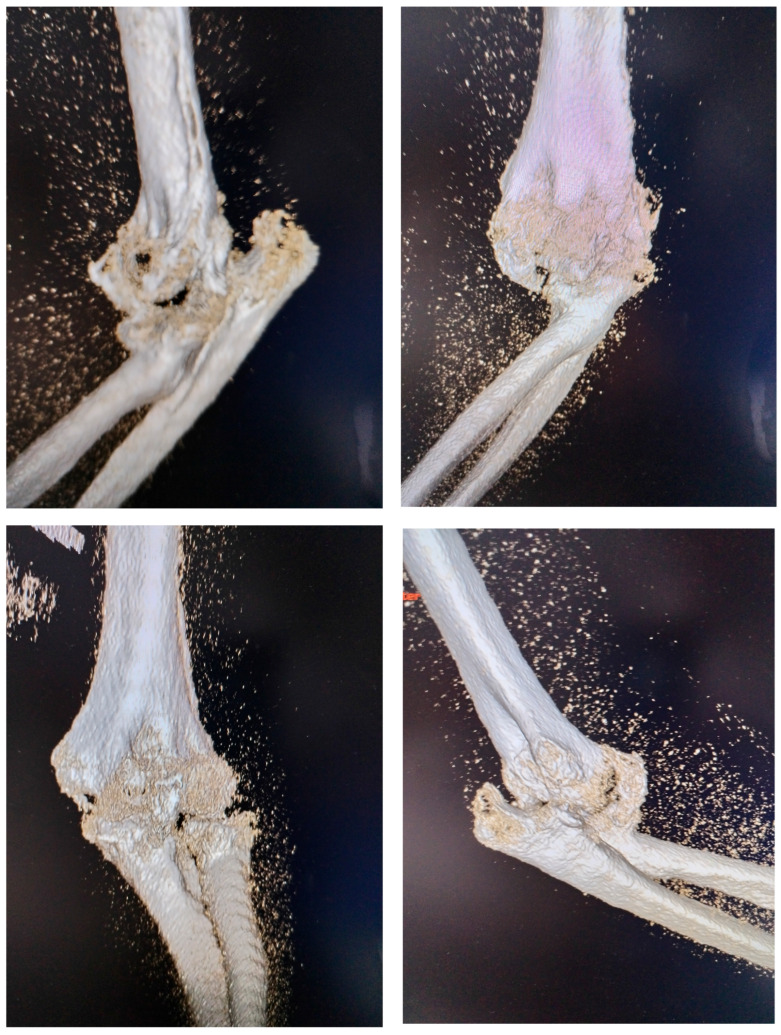
3D CT aspect with visualisation of dislocation and presence of a massive heterotopic bone inside the joint.

At the time of the injury, the posterior elbow dislocation with associated fractures was not fully diagnosed. The patient initially underwent closed reduction and cast immobilization, but the original radiographs taken immediately after the trauma and following the first reduction were unavailable. Radiographs obtained at the time of admission to our clinic were inconclusive due to extensive heterotopic ossification, prompting a 3D computed tomography (CT) scan to better visualize the bony anatomy and the joint relationships (Figure 2). The CT scan revealed a chronic posterior elbow dislocation with a large heterotopic bone mass within the joint. The coronoid process of the ulna was no longer discernible, suggesting resorption or fragmentation at the time of injury. The radial head was severely deformed (Masson 3), preventing normal articulation with the capitulum and functionally blocking the proximal radioulnar joint.

To rule out nerve entrapment or structural lesions, magnetic resonance imaging (MRI) was performed, confirming intact neurovascular structures and corroborating the clinical examination. No further imaging or laboratory tests were considered necessary, as surgical planning could proceed based on the available radiologic and clinical data.

To objectively assess the patient’s baseline functional status and monitor postoperative outcomes, the Mayo Elbow Performance Index (MEPI) was used (Table 2). This system evaluates pain, range of motion, stability, and daily function. The patient’s preoperative MEPI score was 25, indicating very poor function with severe pain, minimal mobility, joint instability, and significant limitations in daily activities. This assessment confirmed the need for surgical intervention aimed at restoring alignment, alleviating pain, and improving overall elbow function.

## 6. Therapeutic Intervention

Managing neglected posterior elbow dislocations is particularly challenging, especially in cases like this one, due to extensive soft tissue contractures, heterotopic ossification, ligamentous insufficiency, fibrosis, fractures, capsular lesions, and the requirement for prolonged postoperative physiotherapy [9]. These issues are compounded by patient frustration and persistent pain. The primary goal of surgical intervention is to restore a pain-free, stable, and functional elbow with congruent joint surfaces.

Closed reduction was not feasible due to bone mass formation and radial head deformity, as well as capsular retraction with a persistently dislocated elbow. Open reduction is generally required to manage such cases. Various surgical approaches have been described, including a single posterior approach [4,10] and a two-incision technique [11]. Each approach has specific advantages and limitations. The posterior approach allows excellent visualization of the joint and contracted soft tissues, facilitating precise manipulation and reduction. When necessary, radial head osteosynthesis or arthroplasty can be performed. To maintain reduction, transfixing the olecranon to the humerus with one or two small smooth pins, with the elbow flexed at 90° for approximately 14 days, is recommended to support soft tissue healing, followed by controlled active physiotherapy [12]. When available, a hinged external fixator is often preferred [13].

In long-standing neglected dislocations, triceps contracture can further complicate surgery [12,14]. Tricepsplasty is indicated when contracture prevents reduction and is typically performed via a midline posterior incision with medial and lateral dissection in the deeper layers [15]. The main drawback of this approach is the potential for joint instability.

The two-incision technique, by contrast, preserves the triceps insertion and has been associated with better functional outcomes compared to the posterior approach [16]. The lateral approach provides superior exposure of the humeroradial joint and anterior coronoid fossa, while the medial approach allows access to the posteromedial capsule for extraperiosteal dissection. A limitation is that tricepsplasty remains difficult without a posterior midline incision, but preserving the triceps offers advantages such as reduced postoperative pain, improved joint stability, enhanced range of motion, and earlier rehabilitation [17].

For this patient, we selected the two-incision approach to maximize postoperative joint stability. Under general anesthesia, the patient was positioned supine with the affected upper limb crossed over the chest, providing optimal exposure for concurrent medial and lateral approaches. A proximal arm tourniquet was applied. The ulnar nerve was identified medially and carefully protected throughout the procedure, as its separation from fibrous and bony tissue was challenging.

**Figure 3:**
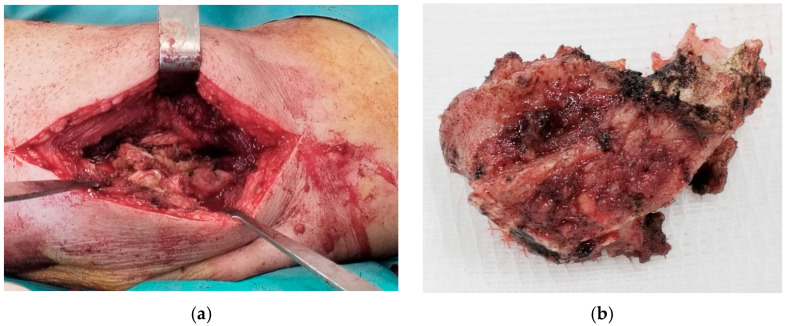
(**a**) Lateral approach; (**b**) massive heterotopic ossification.

Considering the high risk of postoperative instability, we preferred the two-approach technique—lateral and medial—in order to preserve the integrity of the triceps tendon. Based on our experience and the existing literature, this method reduces the risk of joint instability and maintains the muscular strength of the elbow extensor mechanism [12,13,14,15]. The combined medial and lateral approach allowed removal of a large anterior ossification (Figure 3) and cleaning of the posterior compartment, while preserving the triceps insertion to maintain postoperative stability. After releasing all soft tissues from bone, except the triceps insertion, and clearing the articular surfaces of adhesions, the anterior capsule was anchored to the coronoid process using two anchors to prevent posterior dislocation. The common extensor tendons were detached from the distal humerus, as they were retracted and obstructed visualization and removal of intra-articular ossification.

The collateral ligaments were detached to improve joint mobility. Rather than reconstructing them, the ligaments were reattached to adjacent fibrous tissue to avoid excessive tension. The damaged radial head was removed, and radial head arthroplasty was performed to provide additional stabilization (Zimmer Biomet: ExploR Modular Radial Head, cemented type). While osteosynthesis is usually preferred, in this chronic case, the radial head was irreparably damaged and blocked movement. After arthroplasty, pronation and supination were checked to ensure functional mobility.

During elbow manipulation, a small olecranon fragment became displaced and was stabilized with a screw. Post-fixation, the elbow achieved a range of motion of approximately 100–120°, although full extension was limited due to contracture of the biceps and brachialis muscles. Pronation and supination were assessed with the elbow flexed at 90° and were found to be complete.

**Figure 4:**
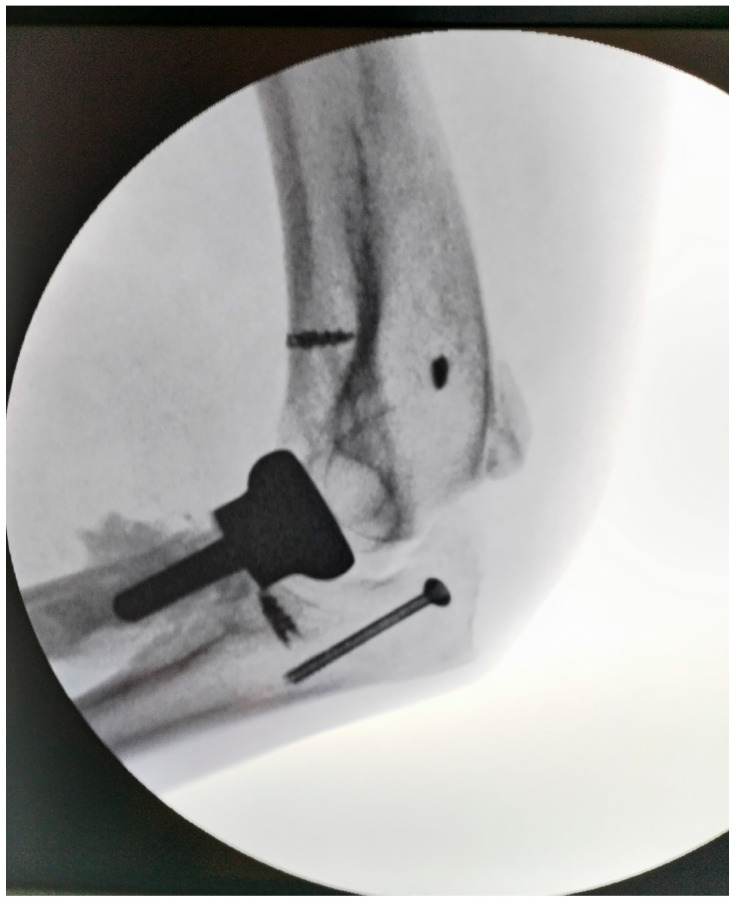
Intraoperative aspect of elbow (radial head arthroplasty and anchors for muscle fixation; the tip of olecranon was stabilized with a cannulated screw).

The common extensor tendons were reattached using anchors, and an anterior transposition of the ulnar nerve was performed. Soft tissues were sutured with the elbow positioned at 90° and the radius in pronation, utilizing the prosthetic radial head as a stabilizer. Postoperative radiographs were obtained to confirm proper joint alignment (Figure 4). The wound was closed in a standard fashion over a suction drain to prevent subcutaneous hematoma formation.

Following surgery, a posterior splint was applied with the elbow at 90° in a neutral position for 2–3 weeks to allow soft tissue swelling to subside [18]. Antibiotics were administered intraoperatively and postoperatively to prevent infection, and local ice therapy was applied for several days to reduce inflammation.

## 7. Follow-Up and Outcome

Surgery represents only part of the overall treatment for elbow injuries. Postoperatively, active finger movements and limb elevation were initiated immediately. Although the arm was immobilized, the splint was removed daily starting on the first day after surgery to allow guided flexion–extension and pronation–supination exercises aimed at preserving elbow mobility. Early mobilization, both gentle passive and active range-of-motion exercises, is essential, as some mobility is often lost immediately after anesthesia due to pain and reflex muscle contraction.

After three weeks, the splint was permanently removed and replaced with a sling. At six weeks, once soft tissue healing was sufficient and a functional range of motion was achieved, the patient began a more intensive rehabilitation program. In theory, the posterior splint could be worn at night for up to three months to prevent dislocation recurrence, and postoperative physiotherapy may need to continue long-term for optimal outcomes. In this case, however, the patient had to interrupt rehabilitation after the first two to three months due to family obligations, resulting in some loss of elbow extension.

Heterotopic ossification is a common postoperative complication but can be prevented with prophylaxis using indomethacin (25 mg three times daily for six weeks with gastric protection), cold packs, and supervised physiotherapy [18]. Other complications, such as wound infection, early osteoarthritis, and nerve injury, are rare when a sterile, careful, and meticulous surgical technique is used, including thorough ulnar nerve release [19].

**Figure 5:**
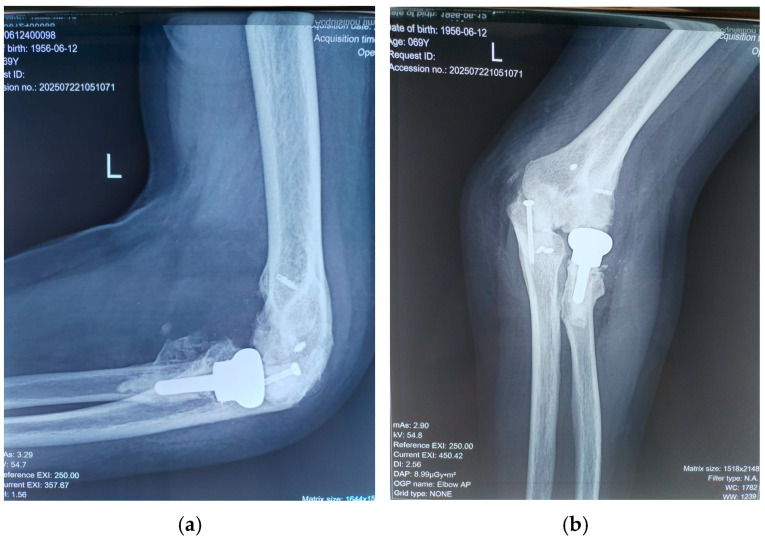
(**a**) Lateral and (**b**) anterior view of elbow at 18 months after surgery.

Sutures were removed at two weeks postoperatively. Follow-up assessments occurred at six weeks, three months, and 18 months, at which point the patient’s MEPI score had improved to 95 (Table 2). Between four and six weeks post-surgery, passive exercises were gradually replaced by active movements. No wound infections or ulnar nerve deficits were observed. Follow-up radiographs at 18 months confirmed maintained reduction, proper positioning of the radial head prosthesis, and no recurrence of heterotopic ossification (Figure 5).

Functional elbow motion typically requires a flexion–extension arc of 75–120° and about 50° of pronation–supination, which is generally sufficient for daily activities [20].

**Figure 6:**
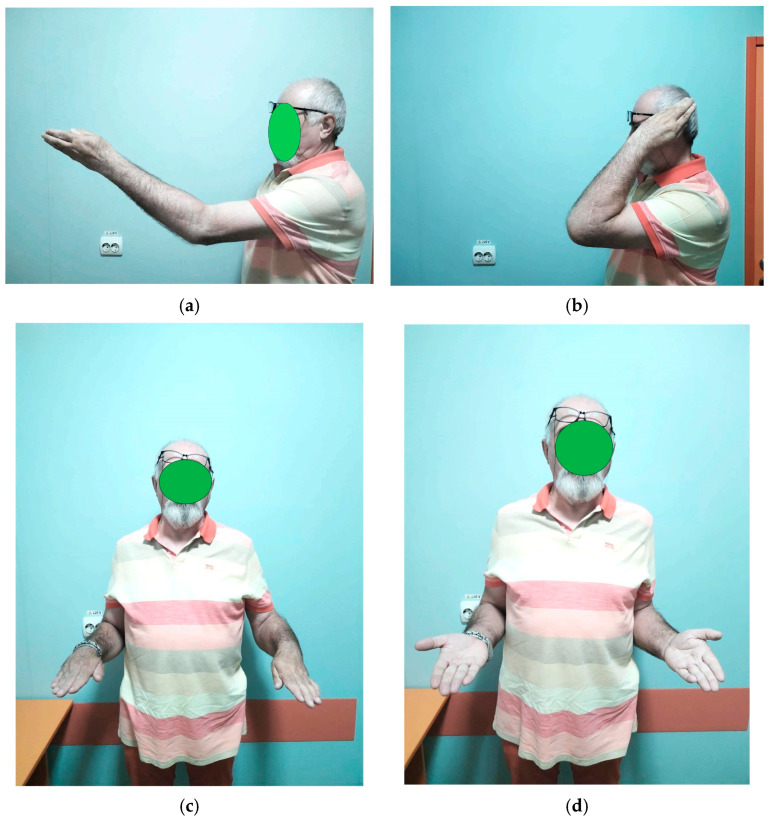
(**a**) Extension, (**b**) flexion, (**c**) pronation, and (**d**) supination of elbow at 18 months after surgery. Photographs were obtained with the patient’s written informed consent for scientific publication.

Our patient presented a stable, nonpainful joint with a range of motion (flexion–extension) of 75° in the morning, with an increase of up to 80° during the day. It is known that after waking up from anesthesia, because of pain and muscular contracture, some degrees of motion are lost; for this reason, rehabilitation must begin early, with most recovery happening between 6 weeks and 3 months after surgery [21]. Although the patient was unable to follow the full prescribed rehabilitation plan due to family reasons, at 18 months post-surgery, he has a pain-free joint, a flexion–extension arc of 80°, and pronation and supination are nearly normal, allowing him to carry out daily activities and work (pronation was 80° and supination was 75°). This mobility and stability of the joint currently allow him to do activities such as washing his face, shaving, dressing, combing his hair, and working. The strength of the forearm was quite good, with the patient being able to carry a few kg without problems (maximum was 12 kg) (Figure 6). No adverse or unanticipated events occurred during the follow-up period. The patient did not experience infection, neurovascular complications, prosthesis loosening, or additional fractures.

## 8. Discussion

This case underscores the inherent complexity in treating neglected posterior elbow dislocations complicated by associated fractures and heterotopic ossification. It was evident that this case was not amenable to conservative management, and surgical intervention represented the only appropriate course of treatment.

Two operative strategies were considered: a posterior approach, which would have necessitated a tricepsplasty—providing excellent articular exposure but compromising the integrity of the extensor mechanism—and a dual (medial and lateral) approach. The latter allowed comprehensive access to both the anterior and posterior compartments of the elbow while preserving the triceps mechanism intact. Given the extended interval since the initial dislocation and the chronicity of the joint malalignment, the dual approach was deemed more advantageous, offering a balance between adequate exposure and preservation of postoperative function [19,20,22,23].

The principal strength of our management strategy was the implementation of a dual-incision surgical approach in conjunction with radial head arthroplasty and meticulous excision of heterotopic bone. This combination facilitated the restoration of both joint stability and functional mobility. Radial head arthroplasty was selected as the sole surgical solution because the post-fracture deformity of the radial head, combined with the prolonged interval since the initial trauma, rendered osteosynthesis unfeasible. Restoration of the radial head anatomy was essential to re-establish mechanical stability of the elbow joint.

However, the absence of initial radiographs and the delayed presentation represented significant limitations, increasing the technical demands of the procedure and constraining the ultimate range of motion achievable.

The existing literature consistently emphasizes that neglected elbow dislocations present major challenges due to soft tissue contracture, ossification, and persistent joint instability [1,2,9,22,23]. Early diagnosis and timely intervention are essential to prevent irreversible stiffness and long-term functional compromise. Prior studies have demonstrated that open reduction, excision of ossifications, and, when indicated, radial head arthroplasty can yield satisfactory outcomes even in chronic cases [5,8,24,25,26,27,28,29]. Our treatment approach was informed by these principles, with an emphasis on preserving joint stability and promoting early postoperative mobilization.

Prevention of heterotopic ossification recurrence was achieved through postoperative administration of indomethacin, 25 mg three times daily for a duration of six weeks.

In conclusion, this case provides valuable evidence supporting the safety and efficacy of a dual-incision surgical approach combined with radial head arthroplasty for the management of complex chronic elbow dislocations with secondary heterotopic ossification. The conclusions are reinforced by the favorable postoperative course: at 18 months of follow-up, the patient demonstrated a stable, pain-free elbow with a functional range of motion. These findings highlight that, even in delayed and complex cases with extensive soft tissue and bony changes, careful surgical planning and execution can restore joint stability, preserve the extensor mechanism, and achieve meaningful long-term functional recovery, thereby contributing significant insight to the existing literature on challenging elbow injuries.

## 9. Patient Perspective

The patient expressed satisfaction with the outcome of the treatment. He reported significant improvement in pain, mobility, and the ability to perform daily activities compared to the preoperative state. Although the rehabilitation process required dedication and regular exercises, he appreciated the gradual recovery of elbow function. The patient also emphasized the importance of being treated in a specialized center with experienced surgeons, which he believes contributed to the positive result.

## 10. Informed Consent

Written informed consent was obtained from the patient for publication of this case report, including all images and clinical data. The patient was informed about the purpose of the publication, the use of anonymized data, and the possibility of dissemination in scientific literature. A copy of the consent form has already been submitted to the journal.

## 11. Conclusions

A fracture dislocation must always be promptly recognized in the emergency room, at which point appropriate treatment should begin: surgical joint exposure, management of the fracture(s), dislocation reduction, and joint stabilization, all followed by intensive rehabilitation starting immediately after surgery. Considering the patient’s severe level of disability and the complexity of the presented case, we believe the postoperative outcome is extremely good (95 MEPI Score) and encouraging for the treatment of such complications.

## Figures and Tables

**Table 1:** Timeline.

Time from Injury	Event
Day 0	Traumatic posterior elbow dislocation with associated radial head and coronoid process fractures.
Weeks 0–3	Initial management in a local hospital: closed reduction and immobilization in a plaster cast.
Months 2–6	Prolonged physiotherapy on a dislocated joint with intra-articular fragments; progressive stiffness and pain.
Month 6	Presentation to our clinic: severe stiffness, deformity, and massive heterotopic ossification confirmed by CT.
Surgery (Month 6)	Open procedure: excision of heterotopic bone, reduction in dislocation, anterior capsule reattachment to coronoid process, and radial head arthroplasty.
Postoperative	Immediate gentle mobilization; supportive splint used at night only.
2 weeks post-op	Suture removal; patient instructed on gentle assisted mobilization.
6 weeks post-op	Progressive active exercises; monitoring for recurrence of ossification; adherence to rehabilitation confirmed.
18-month follow-up	Stable, pain-free elbow with functional range of motion (flexion–extension 80°, pronation 85°, supination 80°).

**Table 2:** Mayo Elbow Performance Index (MEPI) Score.

Assessment	Scores
Pain (Max., 45 points)	None (45 points) Mild (30 points) Moderate (15 points) Severe (0 points)
Range of Motion (Max., 20 points)	Arc > 100 degrees (20 points) Arc 50 to 100 degrees (15 points) Arc < 50 degrees (5 points)
Stability (Max., 10 points)	Stable (10 points) Moderately unstable (5 points) Grossly unstable (10 points)
Function (Max., 25 points)	Able to comb hair (5 points) Able to feed oneself (5 points) Able to perform personal hygiene task (5 points) Able to put on shirt (5 points) Able to put on shoes (5 points)

## Data Availability

The original contributions presented in this study are included in the article. Further inquiries can be directed to the corresponding author.
